# Nitrogen Removal Performance and Microbial Community Structure of IMTA Ponds (*Apostistius japonicus*-*Penaeus japonicus*-*Ulva*)

**DOI:** 10.1007/s00248-024-02378-z

**Published:** 2024-06-03

**Authors:** Daiqiang Chen, Chen Tian, Haiqing Yuan, Wei Zhai, Zhiqiang Chang

**Affiliations:** 1 College of Fisheries and Life Science, Shanghai Ocean University, Shanghai, 201306 People’s Republic of China; 2 State Key Laboratory of Mariculture Biobreeding and Sustainable Goods, Yellow Sea Fisheries Research Institute, Chinese Academy of Fishery Sciences, Qingdao, Shandong 266071 People’s Republic of China; 3 College of Marine Science and Fisheries, Jiangsu Ocean University, Jiangsu, 222005 People’s Republic of China

**Keywords:** Denitrification, Anammox, Nitrous oxide, Mariculture ponds, Stable isotope

## Abstract

Denitrification and anaerobic ammonium oxidation (anammox) are key processes for nitrogen removal in aquaculture, reducing the accumulated nitrogen nutrients to nitrogen gas or nitrous oxide gas. Complete removal of nitrogen from aquaculture systems is an important measure to solve environmental pollution. In order to evaluate the nitrogen removal potential of marine aquaculture ponds, this study investigated the denitrification and anammox rates, the flux of nitrous oxide (N_2_O) at the water–air interface, the sediment microbial community structure, and the gene expression associated with the nitrogen removal process in integrated multi-trophic aquaculture (IMTA) ponds (*Apostistius japonicus*-*Penaeus japonicus*-*Ulva*) with different culture periods. The results showed that the denitrification and anammox rates in sediments increased with the increase of cultivation periods and depth, and there was no significant difference in nitrous oxide gas flux at the water–air interface between different cultivation periods (*p* > 0.05). At the genus and phylum levels, the abundance of microorganisms related to nitrogen removal reactions in sediments changed significantly with the increase of cultivation period and depth, and was most significantly affected by the concentration of particulate organic nitrogen (PON) in sediments. The expression of denitrification gene (*narG*, *nirS*, *nosZ*) in surface sediments was significantly higher than that in deep sediments (*p* < 0.05), and was negatively correlated with denitrification rate. All samples had a certain anammox capacity, but no known anammox bacteria were found in the microbial diversity detection, and the expression of gene (*hzsB*) related to the anammox process was extremely low, which may indicate the existence of an unknown anammox bacterium. The data of this study showed that the IMTA culture pond had a certain potential for nitrogen removal, and whether it could make a contribution to reducing the pollution of culture wastewater still needed additional practice and evaluation, and also provided a theoretical basis for the nitrogen removal research of coastal mariculture ponds.

**Graphical Abstract:**

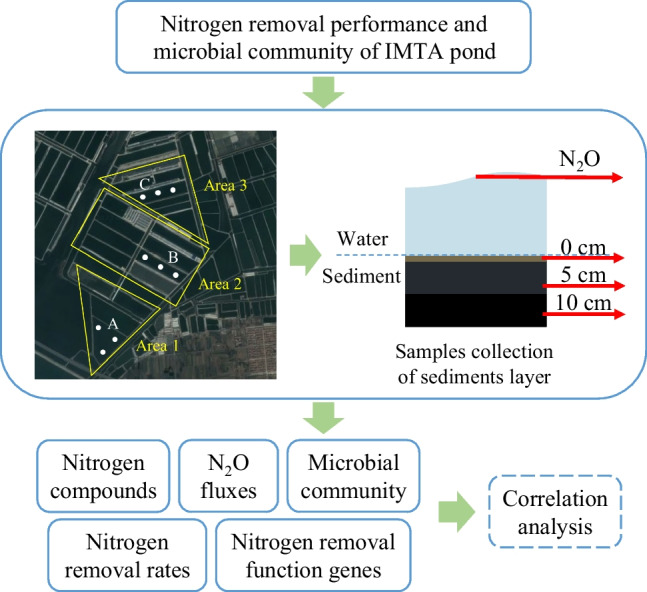

## Introduction

With the expansion of aquaculture scale and the complexity of pond systems, unused nitrogen gradually accumulates in sediments [1]. High concentrations of dissolved inorganic nitrogen (DIN) spread to water volume, leading to eutrophication which has a negative impact on pond water quality and aquatic environment [2]. Nitrogen cycling is a crucial ecological process in aquaculture systems, which is directly related to the quality of aquaculture water and the stability of the ecosystem [3, 4]. Sediments are particularly important for the nitrogen removal in aquaculture systems, because sediments are not only the biogeochemical centers of nitrogen cycling reactions but also the primary habitat of benthic organisms.

In the past few years, studies on the nitrogen removal process of sediments in lakes, estuaries, and offshore seas and their dynamics with microbial communities have gradually become a research hotspot in the field of aquaculture [5–7], but there is less attention to the nitrogen cycling and nitrogen removal of marine aquaculture ponds. The nitrogen removal performance and microbial community structure of pond sediments need to be comprehensively evaluated. Ponds with fish and shrimp as the main breeding species have a certain nitrogen removal ability due to the anaerobic environment formed by a large amount of residual bait deposition [8]. However, it is still unknown whether the ingestion of organic nitrogen in the feeding sediments of sea cucumber breeding ponds has an impact on the nitrogen removal ability of the sediments. Therefore, the nitrogen removal potential and microbial community structure of Integrated Multi-trophic Aquaculture (IMTA) pond sediments with sea cucumber as the main breeding species need to be comprehensively evaluated.

The removal of nitrogen in aquaculture mainly relies on microbial-driven nitrogen removal reactions [9]. Denitrification and anaerobic ammonium oxidation (anammox) are crucial nitrogen removal processes in sediments [10] that reduce the accumulated inorganic nitrogen in sediments to nitrogen gas and completely remove them from the aquaculture system. Denitrification reduces nitrate to nitrous oxide or nitrogen gas, effectively reducing nitrate nitrogen in the system [11], while anammox participates in the nitrogen cycle by oxidizing ammonia nitrogen to nitrogen gas with nitrite as an electron acceptor [12]. Nitrogen oxide (N_2_O) gas is an important greenhouse gas emitted from agricultural production and is a product of denitrification at low temperatures [13, 14]. It is necessary to further understand the relationship between the nitrogen removal rate and the nitrous oxide escape flux at the water–air interface to evaluate the nitrogen removal capacity and greenhouse gas emissions of sediments.

The structure of microbial communities in sediments is closely related to the stability of nitrogen removal reactions and nitrogen cycle [15]. Microorganisms such as denitrifying bacteria and anaerobic ammonium oxidizing bacteria participate in different nitrogen cycle processes, and their relative abundance and activity directly affect the conversion efficiency of nitrogen [16]. The community structure and relative abundance of nitrogen removal-related microbial communities have an important effects on the regulation of sediment nitrogen removal rate, but the relationship between microbial communities and nitrogen removal rate is rarely related. There are significant differences in metabolic activities and the ability to utilize different nitrogen compounds in substrates among different microbial communities, and there are also differences in dominant populations in different ecological environments [17–19]. Therefore, an in-depth understanding of the dynamics of microbial communities in pond sediments and their relation to nitrogen removal rates and the environment is necessary.

The expression of genes related to microbial nitrogen removal is considered as a biomarker for nitrogen cycle-related reaction processes [20–22], and the bacterial population dynamics are evaluated by quantifying gene copy number. The denitrification process includes three related genes *narG*, *nirS*, and *nosZ*, which correspond to the reduction processes of nitrate, nitrite, nitrous oxide, and nitrous oxide [6]. The unique lipids of anaerobic ammonium oxidizing bacteria were used as biomarkers for their presence in the environment in previous studies [21], but now *hzsB* gene is usually used as a biomarker for anammox process [23]. By studying the gene expression of microbial nitrogen removal process, the influence of different environmental conditions on the nitrogen cycle process can be analyzed, so as to better understand the contribution of different microorganisms to the nitrogen cycle and provide a more accurate basis for the nitrogen removal reaction process and change.

The relationship between ecosystem microbial community structure and nitrogen removal performance and environmental factors was currently being explored in an attempt to explain the environmental factors that have the greatest influence on it [24, 25]. In addition, the microbial community structure differs greatly among different aquaculture areas or aquaculture modes, and the dominant nitrogen removal pathways were also different [6, 26]. These basic studies are conducive to understanding of the interaction between microbial community structure and environment in different ecosystems. This study aims to systematically explore the nitrogen removal rate and microbial community dynamics in pond sediments, with special attention to key parameters such as denitrification rate, anammox rate, microbial community structural diversity, and nitrogen removal gene expression, in order to provide a scientific basis for optimizing the nitrogen cycle management of aquaculture system. An in-depth exploration of these aspects is expected to reveal the changing trends of the nitrogen removal process in pond sediments in different environments and provide substantial support for promoting the sustainable development of aquaculture systems.

## Materials and Methods

### Study Area and Sediment Collection

**Fig. 1:**
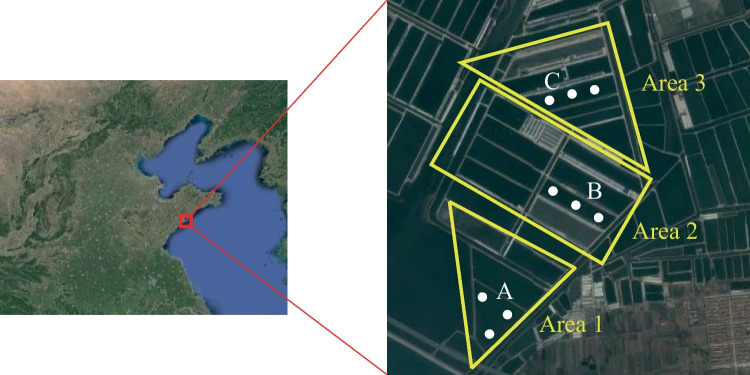
Satellite map of the sites where the samples were collected (Qingdao Ruizi Group Co., Ltd., Qingdao, China). Areas 1, 2, and 3 represent the three rounds of pond cleaning time at 2023, 2022, and 2021, respectively. A, B, and C represent the ponds in 5th, 17th, and 29th months of cultivation period, respectively

The study area was selected for IMTA (*Apostistius japonicus*-*Penaeus japonicus*-*Ulva*) farming ponds in production, which belongs to Ruizi Group Co. Ltd., Qingdao, Shandong province of China (Fig. 1). There are three different rounds of culture ponds at the experimental site, with pond cleaning times of 2023, 2022, and 2021 for areas 1, 2 and 3, respectively. After the ponds were cleaned, each pond was used for 3 years, and the ponds were cleaned every 3 years. Three ponds with different breeding time (5th, 17th, and 29th months) after last pond cleaning were selected to study the effects of different breeding time on sediment nitrogen removal performance and microbial community. Samples of 0 cm, 5 cm, and 10 cm surface deposits were collected in ponds A, B and C, respectively. The aquaculture animals were *Apostistius japonicus* mixed with *Penaeus Japonicus* and *Ulva*, and pond water was taken from the natural seawater of the coastal sea. The water level of the pond was maintained at 2.0 – 2.5 m in summer and autumn with relatively high optical radiation, and at 1.5 – 2.0 m in winter and spring with relatively low optical radiation. Due to the culture pond in this study was not fed and the sediment accumulation rate was 4.1 – 5.5 cm per year (This study), samples were collected per 5 cm from surface sediment. All fresh sediment samples were stored at − 80 °C after mixed sufficiently for subsequent analysis.

### Sediment Nitrogen Compound Parameter Analysis

Fresh samples were collected from the bottom of the pond to determine the concentrations of the main nitrogen compounds in the sediment: particulate organic nitrogen (PON), dissolved organic nitrogen (DON), ammonia nitrogen (NH_4_^+^), nitrite (NO_2_^−^), and nitrate (NO_3_^−^). After centrifuging a sufficient amount of sediment at 2000 r·min^−1^, the supernatant was taken to determine the concentration of dissolved nitrogen compounds. The centrifuged sediment was placed in an oven at 60°C for drying for 24 h, and the particulate organic nitrogen content in the sediment was determined by element analyzer (Germany) after grinding. The total nitrogen, ammonium, nitrite, and nitrate in the pore water were determined separately. Dissolved organic nitrogen is equal to the difference between total nitrogen and dissolved inorganic nitrogen. The determination method was based on the national standard measurement method [27]. The concentration of nitrogen compounds in the sediment was analyzed as an environmental factor to determine the relevance to the rate of nitrogen removal reactions and the structure of the microbial community.

### Nitrous Oxide Gas Collection and Determination

At the same time with sediment collection, static sealed box is used to collect the nitrous oxide gas escaping from the water–air surface in three ponds. The box body is made of transparent PVC, and the height and diameter were 0.5 m and 0.3 m, respectively. The gas samples were collected at 6 h intervals over a 24 h period and N_2_O was quantified utilizing the gas chromatograph (Configuration BID detector, GC-2030, China). N_2_O fluxes were calculated based on ratio of gas concentration to the experimental time, as described in the following equation.

1 $$F=\frac{C}{T}\times \frac{V}{S}\times \rho \times {10}^{3}$$

where *F* (mg m^−2^ day^−1^) denotes the nitrous oxide gas fluxes; *V* (L) and *S* (m^2^) expressed volume and basal area respectively; *C* (mg L^−1^) and *ρ* (g L^−1^) expressed gas concentration and standard pressure density, respectively; *T* (day^−1^) denotes the experiment time of gas sample collection.

### Rates of Denitrification and Anammox Determined by ^15^N Stable Isotope Tracer

The rates of denitrification and anammox of sediments were determined by ^15^N isotope tracer of cultivated samples. Take 30 g fresh sediment sample and 50 mL in-sit filtration of overburden water (0.45 µm filter membrane vacuum filtration), mixed sufficiently to slurry and aerate high purity helium gas (99.99%) for 30 min to remove dissolved oxygen. Take 4 mL slurry with pipette (5 mL) into 12 mL headspace sample bottle (Labco Extainer, Lampeter, UK) and aerate high purity helium for 10 min to exhaust the air in the bottle; then, sample bottles were placed in the oscillator (HY-2 Speed regulating multipurpose oscillator, Jiangsu Province) at the condition of temperature (26 ± 0.5 °C) and light for 5 days cultivated. Each sample was divided into three treatment groups (Ctrl, Amox, Denit); each treatment group has 0 h and 24 h time groups. Grouping and adding isotope were shown in Table 1. Finally, the reactions were terminated by 200 µL ZnCl_2_ (7mol L^−1^) solution in the incubation systems during the experimental period (0 – 24 h). The denitrification and anammox rates were calculated by the production rates of ^29^N_2_, ^30^N_2_, measured by isotope mass spectrometer (GasBenchII-253plus, America), as described in the following equations.

2 $${f}_{29}=\frac{{{\text{P}}}_{{{\text{N}}}_{2}}^{29}}{{{\text{P}}}_{{{\text{N}}}_{2}}^{28}+{{\text{P}}}_{{{\text{N}}}_{2}}^{29}+{{\text{P}}}_{{{\text{N}}}_{2}}^{30}}$$

3 $${f}_{30}=\frac{{{\text{P}}}_{{{\text{N}}}_{2}}^{30}}{{{\text{P}}}_{{{\text{N}}}_{2}}^{28}+{{\text{P}}}_{{{\text{N}}}_{2}}^{29}+{{\text{P}}}_{{{\text{N}}}_{2}}^{30}}$$

4 $${R}_{{\text{amox}}}=\left({f}_{29}-{f}_{30}\right)\times {V}_{a}\times {\left(\frac{{m}_{s}}{{m}_{s}+{m}_{w}}\right)}^{-1}\times {10}^{3}$$

5 $${R}_{{\text{denit}}}=\left({f}_{29}+2\times {f}_{30}\right)\times {V}_{d}\times {\left(\frac{{m}_{s}}{{m}_{s}+{m}_{w}}\right)}^{-1}\times {10}^{3}$$

where *f*_29_ and *f*_30_ denote production of the ^29^N_2_ and ^30^N_2_, respectively; ${\text{P}}_{{\text{N}}_{2}}^{28}\text{,} \, {\text{P}}_{{\text{N}}_{2}}^{29}\text{, }\text{and }{\text{P}}_{{\text{N}}_{2}}^{30}$ expressed ^28^N_2_, ^29^N_2_, and ^30^N_2_ nitrogen contents, respectively; *R*_amox_ and *R*_denit_ respective denote anammox and denitrification rates; *V*_*a*_ and *V*_*d*_ represent the gas volume of the sample bottle in the Amox and Denit treatment groups, respectively; *m*_*s*_ and *m*_*w*_ expressed the weight of water and sediment, respectively.

**Table 1:** ^15^N slurry incubation experiment groups of stable isotope experiments

Groups	Tracer added	Final concentration (μmol·L^−1^)	Isotopes measured
Ctrl	^15^NH_4_^+^	100	^29^N_2_,^30^N_2_
Amox	^15^NH_4_^+^ + ^14^NO_3_^−^	100 + 100	^29^N_2_,^30^N_2_
Denit	^15^NO_3_^−^	100	^29^N_2_,^30^N_2_

### High-Throughput Sequencing for Microbial Analysis

The DNA was first extracted from the sediment sample and then detected by 1% agarose gel electrophoresis. The hypervariable region V3–V4 of the bacterial 16S rRNA gene were amplified with primer pairs 338F (5′-ACTCCTACGGGAGGCAGCAG-3′) and 806R (5′-GGACTACHVGGGTWTCTAAT-3′). The PCR reaction mixture including 4 μL 5 × Fast Pfu buffer, 2 μL 2.5 mM dNTPs, 0.8 μL each primer (5 μM), 0.4 μL Fast Pfu polymerase, 10 ng of template DNA, and ddH_2_O to a final volume of 20 µL. PCR amplification cycling conditions were as follows: initial denaturation at 95 °C for 3 min, followed by 27 cycles of denaturing at 95 °C for 30 s, annealing at 55 °C for 30 s and extension at 72 °C for 45 s, and single extension at 72 °C for 10 min, and end at 4 °C. PCR (TransGen AP221-02: TransStrat Fastpfu DNA Polymerase) amplification specific primers with barcode were synthesized according to the specified sequencing region, and PCR products were tested by 2% agarose gel electrophoresis. Based on the preliminary quantitative results of the electrophoresis, the PCR products were detected and quantified by the QuantiFluor™-ST blue fluorescence quantification system. After the Illumina library was built and used Illumina sequencing, OTU cluster analysis and species taxonomic analysis were performed after the sample was distinguished, and visual analysis such as statistical analysis of OTU-based microbial community structural diversity and significance tests of community composition differences were performed.

### Quantification of the Genes Associated with Denitrification and Anammox

Real-time PCR was used to detect the expression of genes related to nitrogen removal in the samples, including four functional genes: nitrite reductase (*nirS*), nitrate reductase (*narG*), nitrous oxide reductase (*nosZ*), and hydrazine synthase β subunit (*hzsB*). Each sample was quantitated three times in parallel, with primer sequences and reaction conditions according to the reference (Table 2). As shown in the table, two primers were designed for each gene in the pre-experiment, and the electrophoretic gel images under specific conditions were a single band, and a better primer was selected for subsequent formal experiments. After PCR amplification, product purification, cloning vector connection, and plasmid extraction, the absorbance value of the plasmid was determined to prepare the standard curve. After DNA extraction from sediment samples, quantitative results were obtained by fluorescence quantitative PCR instrument (ABI 7300, Applied Biosystems, America).

**Table 2:** Primers used in this study and the corresponding reaction profiles

Specificity	Primer	Sequence (5′-3′)	Thermal profiles
*nirS* gene (qPCR)	CYC_nirS-F	CACGGYGTBTGCGCAAGGGCGC	3 min at 95 °C, 35 cycles consisting of 30 s at 95 °C, 30 s at 58 °C and 60 s at 72 °C
	CYC_nirS-R	CGCCACGCGCGGYTCSGGGTGGTA	
*nosZ* gene (qPCR)	CYC_nosZ-F	CGYTCTTCMTCGACAGCCAGT	
	CYC_nosZ-R	CATGTGCAGNGCRTGGCAGAA	
*narG* gene (qPCR)	LIY_narG-F	TCGCCSATYCCGGCSATGTC	
	LIY_narG-R	GAGTTGTACCAGTCRGCSGAYTCSG	
*hzsB* gene (qPCR)	hszB_396-F	ARGGHTGGGGHAGYTGGAAG	
	hszB_742-R	GTYCCHACRTCATGVGTCTG	

## Results

### Concentration of Nitrogen Compounds in Sediments

The concentrations of the five nitrogen compounds were measured at different culture period and sediment depths, as shown in Table 3. The PON of surface sediments in three different culture period ponds were significantly higher than deeper layer (*p* < 0.01), and decreased with the increase of depth and period. The DON of 29th month was significantly higher than 5th and 17th months, and decreased with increasing depth. The concentration of ammonium and nitrite fluctuated between 12.01–19.47 and 0.11–0.17 mg·L^−1^, respectively, with no significant difference in time and depth. The nitrates of 17th and 29th months were higher than 5th month (*p* < 0.05), and increased with time, but had no significant relationship with depth.

**Table 3:** The environment factors of different depth and period sediments in culture ponds

Samples	Temperature (°C)	pH	PON (mg·kg^−1^)	DON (mg·L^−1^)	NH_4_^+^-N (mg·L^−1^)	NO_2_^—^N (mg·L^−1^)	NO_3_^—^N (mg·L^−1^)
A 0	25.4	8.07	1513.66 ± 176.87	23.17 ± 3.33	18.46 ± 2.03	0.14 ± 0.02	53.99 ± 2.81
A 5	25.4	8.07	371.63 ± 87.28	20.18 ± 2.75	17.12 ± 2.40	0.15 ± 0.03	82.01 ± 8.55
A 10	25.6	8.23	328.94 ± 25.55	14.49 ± 3.52	15.41 ± 1.33	0.13 ± 0.02	74.08 ± 5.27
B 0	26.3	7.93	1212.62 ± 111.55	17.20 ± 2.96	19.47 ± 2.26	0.15 ± 0.02	76.01 ± 3.59
B 5	25.8	8.11	441.12 ± 41.08	17.66 ± 2.46	13.14 ± 2.36	0.15 ± 0.02	105.7 ± 11.18
B 10	26.3	8.03	256.37 ± 27.73	15.92 ± 4.79	17.68 ± 1.69	0.16 ± 0.02	91.69 ± 6.92
C 0	26.7	8.21	1054.37 ± 124.19	43.69 ± 3.91	12.01 ± 1.28	0.17 ± 0.03	115.8 ± 9.32
C 5	26.1	8.05	308.92 ± 32.29	34.55 ± 2.68	12.75 ± 2.30	0.11 ± 0.02	109.6 ± 7.66
C 10	26.4	8.26	441.41 ± 43.5	31.34 ± 2.66	18.12 ± 1.32	0.14 ± 0.02	92.53 ± 8.72

### Rates of Denitrification and Anammox of Different Culture Periods and Depth

**Fig. 2:**
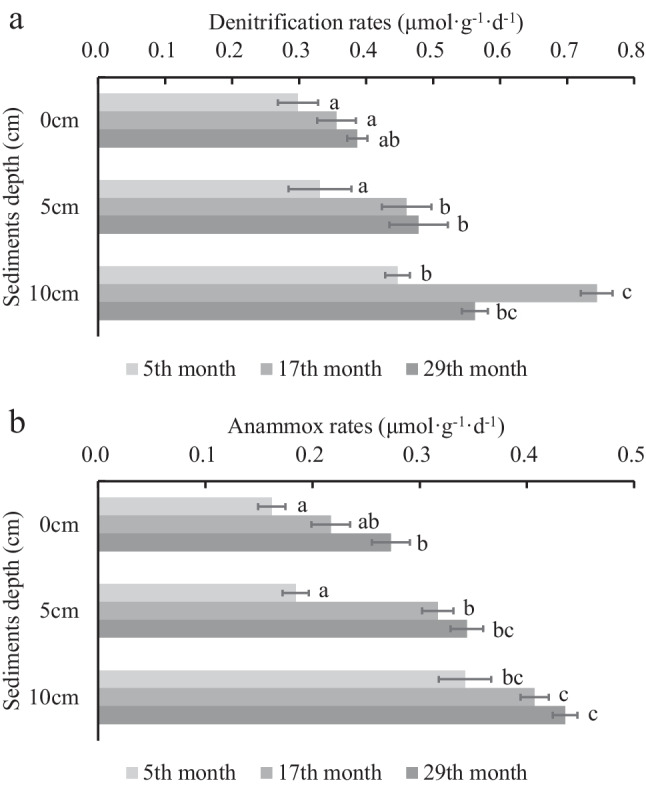
Denitrification and anammox rates of different culture period and depth of sediments

The rates of sediments denitrification and anammox were calculated by measuring the production rates of nitrogen gas, and the results showed that denitrification and anammox rates were fluctuated between 0.29 to 0.74 μmol·g^−1^·day^−1^ and 0.16 to 0.46 μmol·g^−1^·day^−1^, respectively (Fig. 2). Denitrification and anammox rates were converted to nitrogen gas production rates were 4.93 to 12.59 μL·g^−1^·day^−1^ and 4.12 to 10.34 μL·g^−1^·day^−1^, respectively. Rates of denitrification in 5th, 17th, and 29th months both increased with depth, and the rapidly increased rate was in 17th months (*p* < 0.05). The anammox rates also basically increased with depth and cultured period, but the rates of 17th and 29th months were significantly higher than 5th month (*p* < 0.05).

### Nitrous Oxide Gas Fluxes at Water–Air Interface

**Fig. 3:**
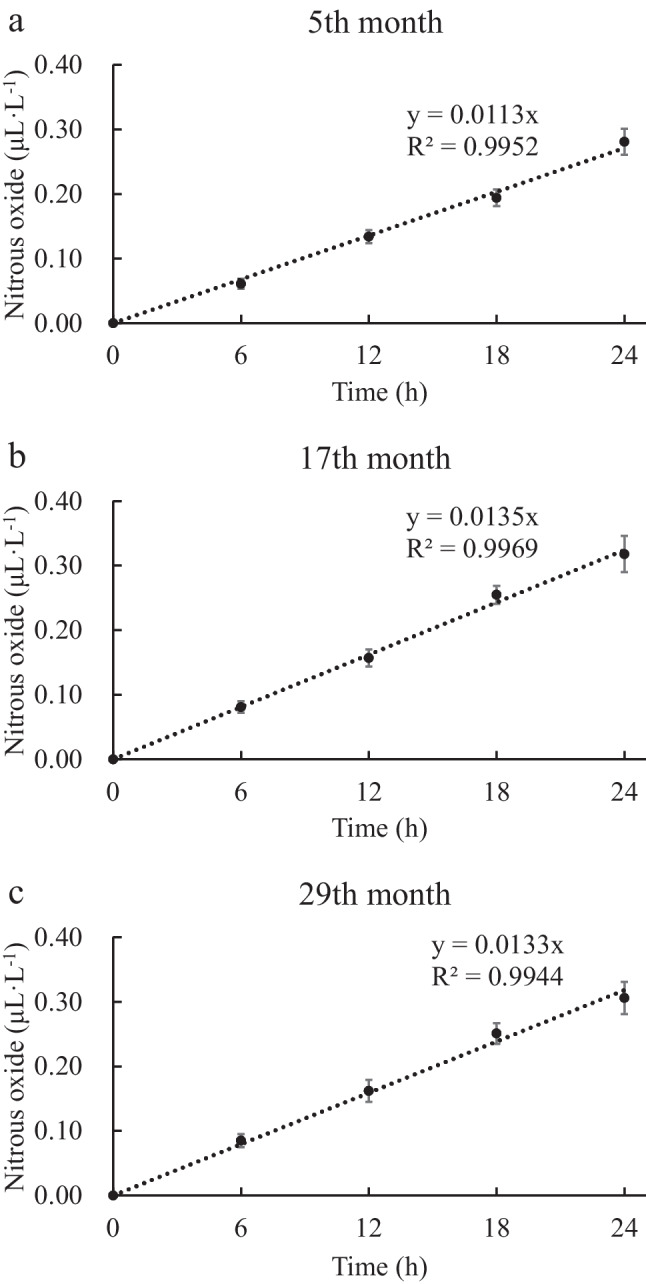
The results of nitrous oxide gas fluxes in 24 h period at water–air interface of culture ponds

Figure 3 shows the time-dependent nitrous oxide gas concentration in the box at the water–air interface of the three ponds. The gas escape rates calculated from the gas masses are 0.602, 0.625 and 0.550 mg·m^−2^·day^−1^, respectively. There is no significant difference in gas escape rates among the three ponds (*p* < 0.05). The mean annual flux of gas in the pond is 216.17 mg·m^−2^·year^−1^. Denitrification is considered to be the main reaction responsible for the production of nitrous oxide gas, while anammox processes reducing nitrite and ammonia to nitrogen gas directly without producing nitrous oxide. Pearson correlation analysis of denitrification rate and gas production showed that 5th month was positively correlated with surface layer, and 5 cm and 10 cm were negatively correlated, 17th month was most correlated with 0 cm and 5 cm, and both were positive correlated, 29th month was significantly negatively correlated with depth, and 5th month was not significantly correlated with depth, and the gas fluxes of 17th month and 29th month were most affected by sediment denitrification rates of 0 cm and 5 cm.

### Microbial Community Structure

High-throughput sequencing was used to analyze the composition structure and changes in microbial communities in different sediment samples. Table 4 shows the concentration of DNA, number of OTUs, coverage, and alpha diversity index of microbial diversity in sediment samples. The minimum coverage is 0.97, indicating that the sequencing results are credible. The maximum and minimum OTU numbers of the sediment samples were A10 and A0, respectively. The OTUs of the 5th month increased with depth, but the 17th month showed the opposite trend, and the 29th month showed no significant change.

**Table 4:** Concentration of DNA, OTUs, and alpha diversity index of total bacterial 16S rRNA gene of pond sediment samples

Sample	Concentration of DNA	OTUs	Shannon	Simpson	Ace	Chao1	Coverage
A 0	13.51	3043	5.96	0.0099	4018	3868	0.98
A 5	15.87	3787	6.41	0.0095	4906	4791	0.98
A 10	13.88	4211	6.65	0.0059	5411	5286	0.97
B 0	8.19	4070	6.63	0.0051	5238	5105	0.97
B 5	5.40	3908	6.58	0.0052	5009	4871	0.98
B 10	7.64	3273	6.31	0.0061	4173	4091	0.98
C 0	17.88	3393	6.03	0.0145	4331	4140	0.98
C 5	14.00	3296	6.22	0.0082	4284	4167	0.98
C 10	10.20	3290	6.30	0.0067	4185	4062	0.98

The identifiers A, B, and C represent the ponds in 5th, 17th, and 29th months of cultivation, respectively; 0, 5, and 10 represent sample collection from sediment depths of 0 cm, 5 cm, and 10 cm; the unit of DNA concentration is ng·μL^−1^

**Fig. 4:**
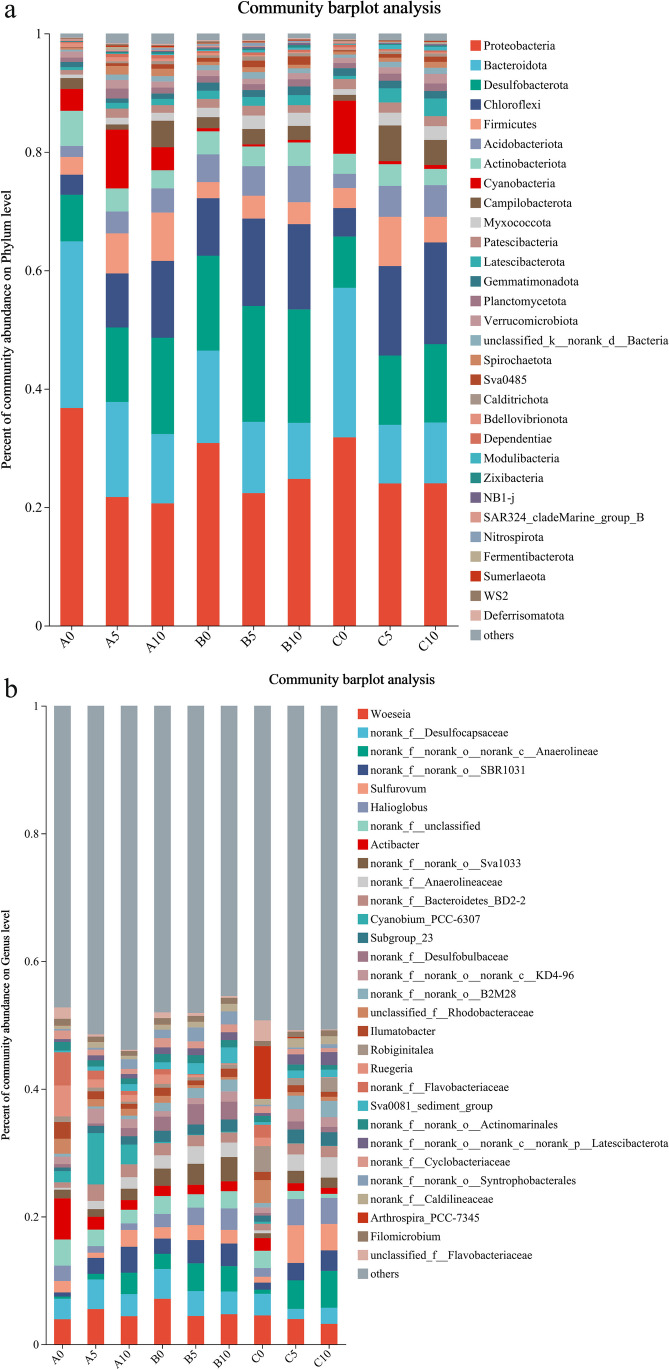
The distribution of bacterial community at phylum level (**a**) and genus level (**b**) of sediment samples

**Fig. 5:**
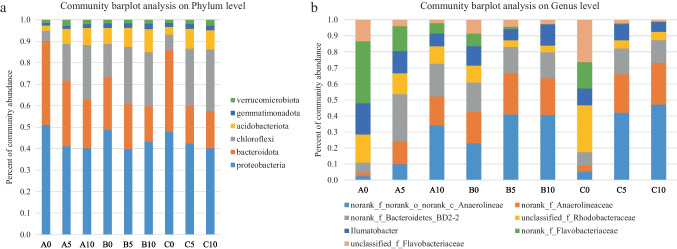
The distribution of bacterial community of denitrification and anammox processes relevant at phylum level and genus level

At the phylum level, the Proteobacteria, Chloroflexi, and Acidobacteria are the most abundant which proportion ranges are fluctuate between 60% and 75%, which include most species of denitrification-related bacteria. While phylum Proteobacteria contains the majority of microorganisms capable of performing heterotrophic denitrification. At the same time, anammox-related bacteria only belong to the Planctomycetota, which the relative abundance fluctuates between 0.007% and 0.016% (Fig. 4a). At the genus level, Illumatobacter and some no rank genus of Anaerolineae, Anaerolineaceae, Rhodobacteraceae, and Flavobacteriaceae contain many common denitrifying bacteria [28], but no anammox-related bacteria were found in the top 30 relative abundance of communities (Fig. 4b). The anammox process consists of six genera, *Candidatus* Anammoxoglobus, *Candidatus* Brocadia, *Candidatus* Scalindua, *Candidatus* Jettenia, *Candidatus* Kuenenia, and *Candidatus* Anammoximicrobium [29]. The relative abundances of denitrification–related and anammox-related genera were re-calculated, as shown in Fig. 5. The relative abundance of most species of denitrifying bacteria changed obviously with the increase of sediment depth or culture time, and the microbial community structure reflected the progressive relationship between different depths and culture time.

**Fig. 6:**
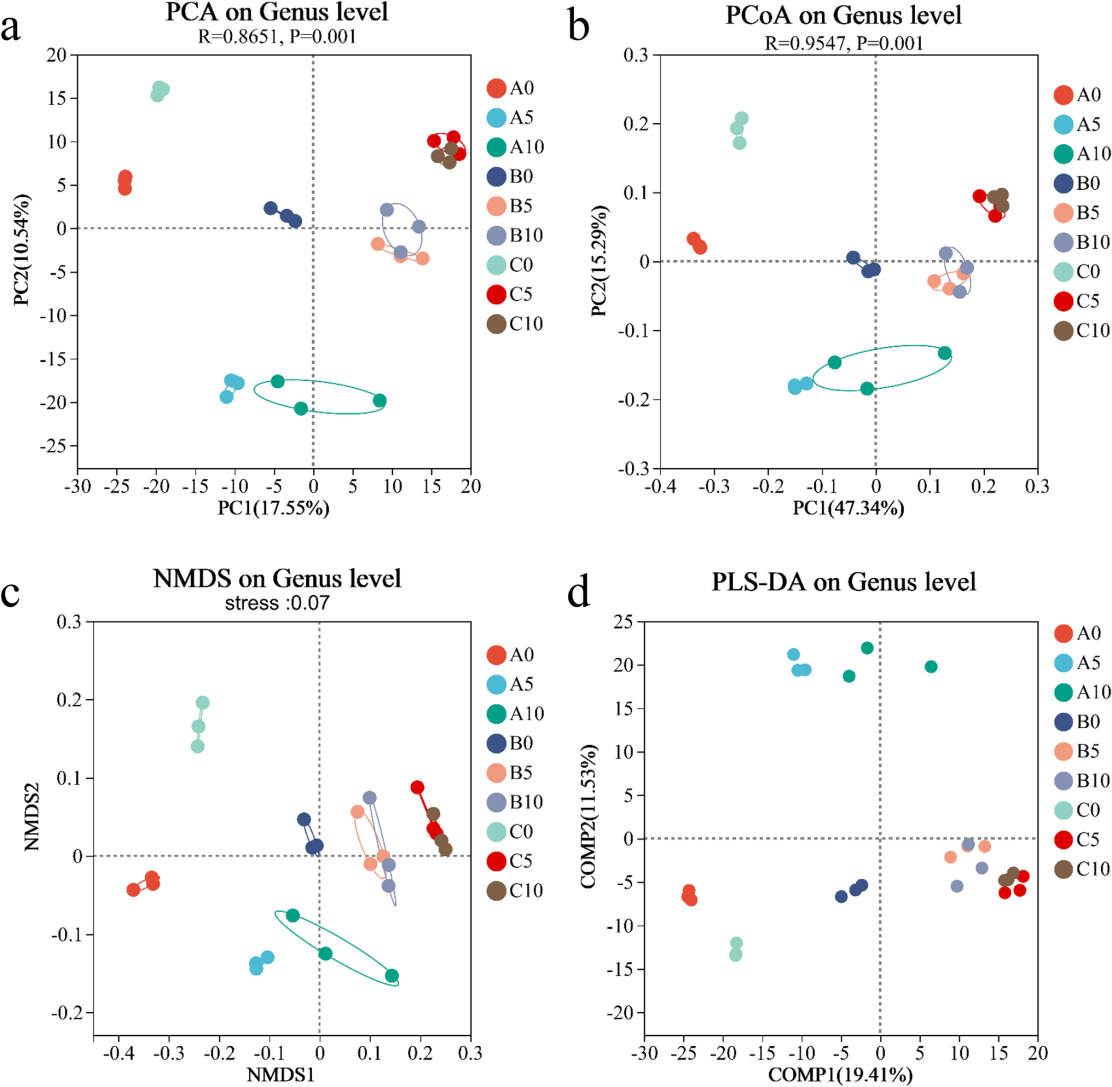
The PCA, PCoA, NMDS, and PLS-DA analysis of bacterial community on genus level

The species diversity of different microbial communities was compared and analyzed by scatterplots to explore the similarity or difference of community composition among samples of different groups (Fig. 6). The results showed that there were significant differences between the microbial communities of surface and deeper layer sediments in different culture periods, but no significant differences between groups 5 cm and 10 cm. The analysis results on OTU level showed that significant differences between the A0 and B0 groups, but the genus level indicates that there are different degrees of intra-group differences. Microbial community composition and diversity analysis can be used to assess differences between different groups and repeated differences within the same group, and to infer differences at parallel sampling points across the pond.

### Dynamics of Nitrogen Functional Genes

**Fig. 7:**
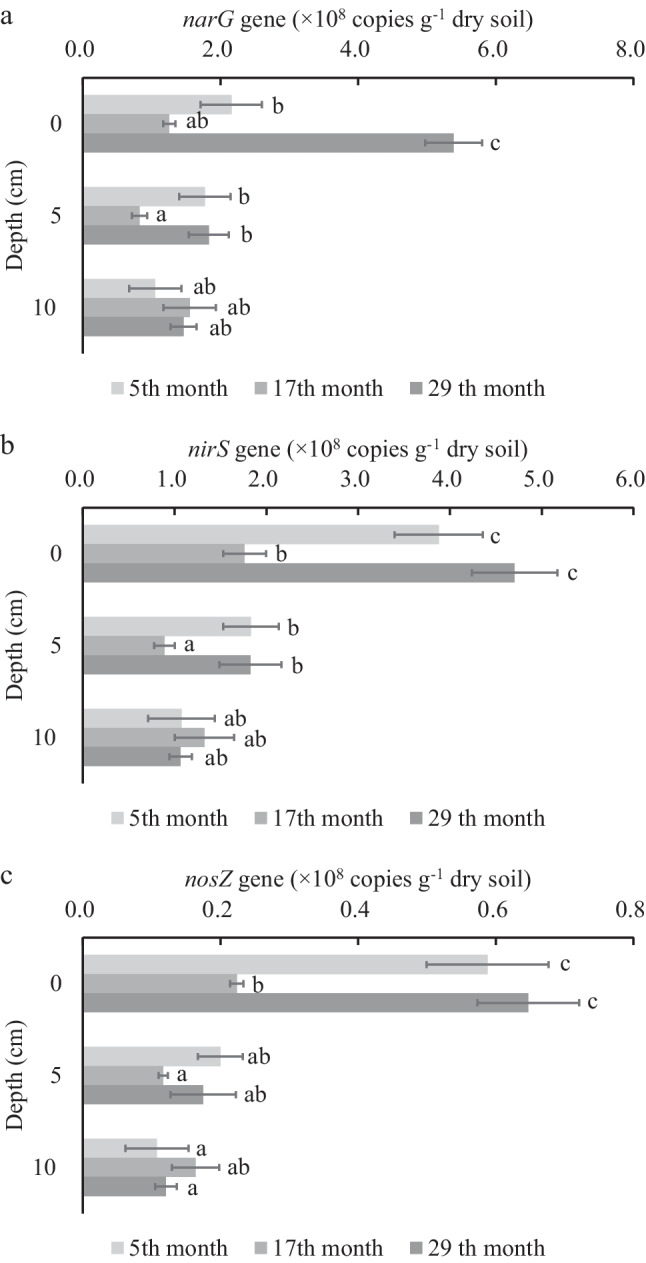
Abundances of nitrogen removal functional genes in sediments: *narG* (**a**), *nirS* (**b**), and *nosZ* (**c**).Error bars indicate standard deviation (*n* = 3)

The absolute quantitative results of the four genes are shown in the Fig. 7, and the copy number of the three genes (*narG*, *nirS*, *nosZ*) related to denitrification is 0.11–5.38 × 10^8^ g^−1^ dry soil. The gene copy numbers in the surface sediment of pond A and pond C were significantly higher than that of other samples (*p* < 0.01), with the gene copies of 3.87-5.38 × 10^8^ g^−1^ dry soil, and the copies range of other samples fluctuates between 0.11 - 2.15 × 10^8^ g^−1^ dry soil. The copy number of *hszB* gene related to anammox was not detected, indicating that there are no anammox reaction microorganisms in the sediment. However, the results of the anammox rate determination by isotope tracer indicate a degree of anammox in the sediment, an anomaly that requires further explanation.

### Correlation Analysis Between Nitrogen Compounds and Nitrogen Removal Processes

Correlations between the concentrations of five nitrogen compounds and nitrogen removal rates, diversity indexes, microbial community composition, and other reaction processes were analyzed to infer the potential impact of nitrogen compounds on nitrogen removal. Pearson correlation analysis showed that DON had the greatest effect on the diversity index (*p* < 0.01), and only nitrite had a positive correlation on the diversity index (Table 5). There was a significant negative correlation between PON and denitrification (*p* < 0.05), a significant positive correlation between nitrate (*p* < 0.05), and a significant negative correlation between nitrite and nitrate on anammox (*p* < 0.05), while other nitrogen compounds had no significant effect (Table 6).

**Table 5:** Pearson correlations between nitrogen compounds and total bacterial alpha diversity in sediment samples of culture ponds

Nitrogen factors	Simpson	ace	Chao	Shannon	Coverage
Temperature	0.092	− 0.197	− 0.210	− 0.111	0.244
pH	0.365	− 0.077	− 0.075	− 0.196	0.068
PON	0.327	− 0.157	− 0.191	− 0.401*	0.091
DON	0.615**	− 0.383*	− 0.412*	− 0.530**	0.379
Ammonium	− 0.021	− 0.429*	− 0.401*	− 0.286	0.416*
Nitrite	0.108	0.050	0.038	0.041	− 0.006
Nitrate	0.235	− 0.102	− 0.108	− 0.036	0.162

*PON* particle organic nitrogen, *DON* dissolved organic nitrogen

*Correlation is significant at the 0.05 level. **Correlation is significant at the 0.01 level

**Table 6:** Pearson correlations between nitrogen compounds and anammox and denitrification rates in sediment samples

	Temperature	pH	PON	DON	Ammonium	Nitrite	Nitrate
Anammox rate	− 0.024	0.208	0.333	0.103	0.109	− 0.414*	− 0.326
Denitrification rate	0.258	0.158	− 0.635**	0.171	0.095	− 0.128	0.416*

*Correlation is significant at the 0.05 level. **Correlation is significant at the 0.01 level

**Fig. 8:**
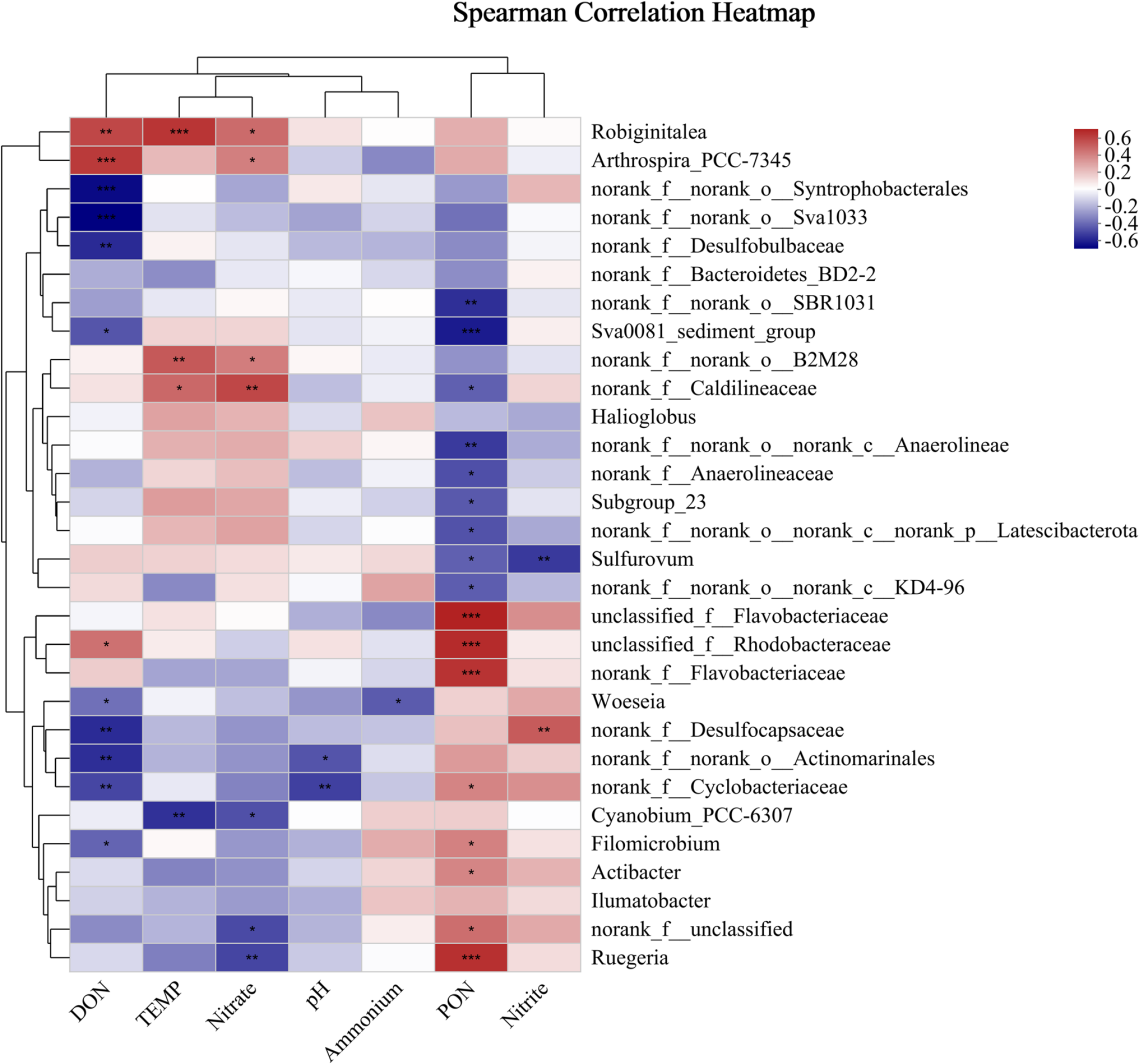
Spearman correlations heatmap analysis of different depth and culture period in sediment and nitrogen compounds at genus level. *Correlation is significant at the 0.05 level. **Correlation is significant at the 0.01 level. ***Correlation is significant at the 0.001 level

**Fig. 9:**
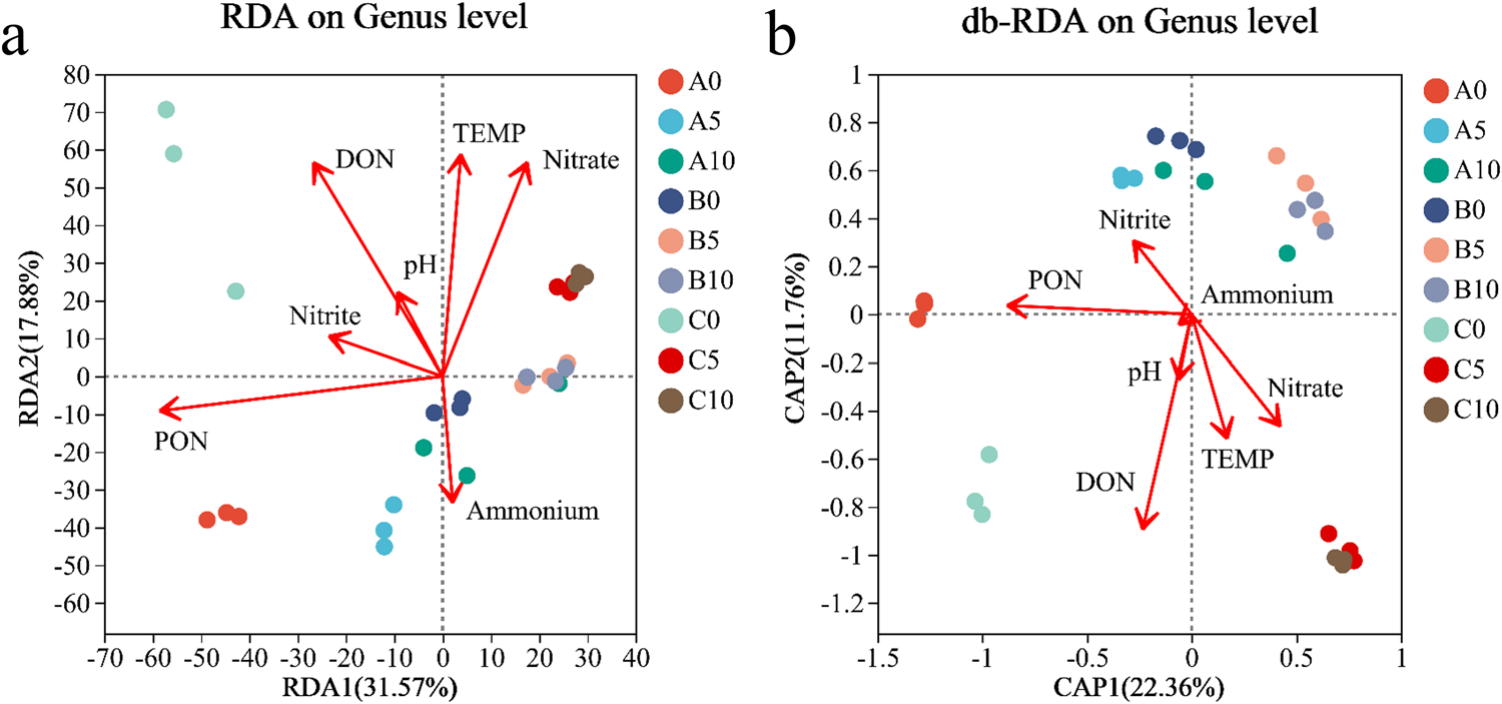
RDA analysis of different depth and culture period in sediment and nitrogen compounds at genus level

Pearson correlation heatmap analysis results showed that PON was significantly positively correlated with Proteobacterota on phylum level while significantly negatively correlated with Firmicutes, Acidobacteriota, and Chloroflexi, and other nitrogen compounds had no significant effect on main denitrification microorganisms. At the genus level, PON was negatively correlated with one kind of Anaerolineae, positively correlated with Flavobacteriaceae and Rhodobacteraceae, and positively correlated with DON on Rhodobacteraceae (Fig. 8). Similarly, other compounds had no significant effect on main denitrification of microorganisms. The results of RDA analysis, which was used to reflect the relationship between bacterial and environmental factors, showed that the difference was significantly positively correlated with the largest A0 and C0 groups in other groups by PON and DON, while the other groups were significantly positively correlated with ammonium and negatively correlated with other nitrogen compounds (Fig. 9).

The analysis of the correlation between the number of nitrogen-removal-related gene copies and the rate of nitrogen-removal reactions with nitrogen compounds is shown in Table 7. PON and DON were significantly positively correlated with denitrification-related gene copy numbers, but the denitrification reaction rates were significantly negatively correlated with gene copy numbers (Table 8).

**Table 7:** Pearson correlations between nitrogen compounds and gene copies of nitrogen removal processes in sediment samples

	*nirS*	*nosZ*	*narG*
Temperature	0.205	0.162	0.442*
pH	0.068	0.025	0.017
PON	0.707**	0.770**	0.387*
DON	0.547**	0.474*	0.740**
Ammonium	− 0.109	− 0.056	− 0.219
Nitrite	0.202	0.252	0.365
Nitrate	− 0.024	− 0.108	0.372

*Correlation is significant at the 0.05 level. **Correlation is significant at the 0.01 level

**Table 8:** Pearson correlations between rates and gene copies of nitrogen removal processes in sediment samples

	*nirS*	*nosZ*	*narG*	*hszB*
Anammox rate	− 0.011	0.071	− 0.232	0.065
Ddenitrification rate	− 0.522**	− 0.549**	− 0.217	− 0.433*

*Correlation is significant at the 0.05 level. **Correlation is significant at the 0.01 level

## Discussion

In this study, denitrification accounted for 57 – 65% of the total nitrogen production in seawater pond sediments, and anammox accounted for 35 – 43% of the nitrogen production, indicating that denitrification contributed the most to sediment nitrogen removal. Existing studies have shown that the contribution of anammox to nitrogen production in surface rice soil in southern Chin is 0.6 – 15% in southern China [19], and the contribution of denitrification to nitrogen production is 10 – 150 times that of anammox [30]. In coastal beaches, tidal flat wetlands, and other terrestrial ecosystems, it has also been shown that denitrification plays a leading role in the nitrogen removal process in soil [31, 32]. However, in some marine ecosystems, the contribution rate of anammox to nitrogen removal exceeds that of denitrification [33, 34], which is contrary to the results of terrestrial ecosystems. In this study, the contribution of denitrification in coastal land-based pond ecosystems was 60% and it can be inferred that the dominant role of denitrification in nitrogen removal gradually shifts to anammox during the transition from terrestrial to marine ecosystems.

This study indicates that the apparent anammox rate and denitrification rate of sediments in seawater ponds increase with depth and cultured time, and the structural levels of microbial communities such as Chloroflexi and Acidobacterota also show similar tendency, instead of the trends of Proteobacteria and Bacteroidota are reverse. The species associated with denitrification also tended to increase or decrease with depth and culture period. Anammox bacteria are considered to be endemic to different ecological environments, with *Candidatus* Brocadia and *Candidatus* Kuenenia being the dominant species in terrestrial soil and freshwater ecosystems [35] and *Candidatus* Scalindua being the dominant species in marine ecosystems [36–38]. Although we detected a degree of anammox reaction rates in the nitrogen removal rate determination experiment, no currently dominant species of anaerobic ammonium-oxidizing bacteria was found in the microbial community structure analysis of all samples (Fig. 4). The reason for this may be the presence of unidentified or unclassified anaerobic ammonium-oxidizing bacteria in transitional environments from terrestrial to marine ecosystems, which may be a new discovery. Another possible reason was the failure to extract the DNA from anaerobic ammonium-oxidizing bacteria due to operational error, but the results of our repeated experiments are also the same. The quantitative results on genes in this study showed that the *hszB* gene associated with the anaerobic ammonium-oxidizing reaction is almost undetectable, with extremely weak electrophoretic bands and few copies of the gene, which also seems to demonstrate the absence of relevant microorganisms. Previous studies have shown that the growth and metabolism of anaerobic ammonium oxidizing bacteria are greatly inhibited by oxygen [39], which can explain the phenomenon of weaker anammox rate and smaller bacterial abundance at the surface. However, stronger anammox reaction was observed at the water–sediment interface in Baiyangdian [40], which may be due to the formation of anaerobic environment caused by the decomposition of organic matter at the surface of sediments, but there was no increase in anammox in deeper sediments.

Studies have shown that the denitrification community structure and related gene (*narG*, *nirS*, *nosZ*) copy number are positively correlated with N_2_O production potential, but negatively correlated with in situ escape gas flux [41], which is similar to the results in this study that N_2_O gas flux is negatively correlated with denitrification rate and gene copy number. Some studies have found that the abundance and diversity of *nosZ* branch are not related to denitrification rate [42], and other research results have also failed to establish the relationship between *nosZ* abundance or diversity and denitrification rate [43, 44]. Since the denitrification process consists of multiple successive reaction steps, and relevant genes such as *narG*, *nirS*, *nosZ*, and *hszB* correspond to different reaction processes, there may be no clear correlation between a gene abundance, microbial diversity, and denitrification rate. The potential generation rate of N_2_O is mainly significantly correlated with environmental factors such as water content, particulate organic matter, and ammonia nitrogen in sediments [45], but the actual N_2_O flux at the water–sediment interface and water–air interface is not significantly correlated with the potential generation rate of N_2_O [41], which may be related to the influence of reaction substrate concentration or sediment pores on gas escape diffusion.

Water content, temperature, salinity, pH, Eh, and nitrogen compound concentration in sediments have varying degrees of influence on the denitrification reaction [46, 47]. The water content in sediments will directly affect the diffusion of oxygen to the bottom, which is one of the important factors regulating denitrification [48].Heterotrophic denitrifying bacteria have higher metabolic activity in an environment abundant in organic matter [49], and denitrifying bacteria tend to be relatively low C/N conditions [50], but for anammox reaction is exactly the opposite [51]. This partly explains the significant positive correlation of high concentrations of organic nitrogen in this study with the expression of genes involved in the denitrification process, but a significant negative correlation with the denitrification rate, which may be due to the high concentration of organic carbon. The concentration of NH_4_^+^ and NO_3_^−^ plasma in the environment will affect the activity of *nosZ* gene and the balance of N_2_O generation and reduction process [52], but in this study, no significant relationship between ammonia nitrogen, nitrite and nitrate, and denitrification-related genes was found. The anammox reaction is primarily influenced by pH. Anammox activity is significantly higher in soils with pH 8.0 – 8.64 than in soils with pH 6.78 – 7.23, and lower in soils with pH 5.97 – 6.01; there is no anammox reaction and *hzsB* gene expression under acidic conditions [30]. Pond cleaning treatments significantly improve the bottom material, allowing adequate oxidation and decomposition of organic matter, and also affect the microbial community structure, which shows a regular trend with increasing culture time and sediment depth.

At present, wastewater discharges and pollution are relatively severe. Taking the open mixed-culture pond in northern Sweden that feeds on municipal sewage as a reference [53], we are considering the possibility of using sea cucumber IMTA pond as a sewage treatment system. Sea cucumbers and shrimp can fed by particulate organic matter in water and sediment, and phytoplankton and microalgae can also absorb ammonia nitrogen and nitrate in water. At the same time, the possible presence of anammox bacteria in the sediment also requires further study to isolate and identify if it is an unknown bacterium, which could be a new discovery.

## Conclusion

In this study, the potential nitrogen removal capacity and microbial community structure of sediments were analyzed to determine the gradient relationship with different culture period and sediment depths. At the same time, some environmental factors and nitrogen compounds were analyzed to determine the interaction of nitrogen elements on nitrogen removal rate and microorganisms in the aquaculture production. In summary, in-depth study of nitrogen removal performance and microbial community dynamics of pond sediments is an important part of promoting sustainable development of aquaculture systems. The nitrogen removal reaction driven by microbial community is closely related to environmental factors.

## Data Availability

No datasets were generated or analysed during the current study.

## References

[1] Kolath AS (2021). Biodiversity and sediment contamination in wet stormwater ponds depending on design and catchment characteristics. Sustainability.

[2] Ke X (2018). Toxicity assessment of sediments from the Liaohe River Protected Area (China) under the influence of ammonia nitrogen, heavy metals and organic contaminants. Environ Toxicol Phar.

[3] Kuypers MMM, Marchant HK, Kartal B (2018). The microbial nitrogen-cycling network. Nat Rev Microbiol.

[4] Sauthier N, Grasmick A, Blancheton JP (1998). Biological denitrification applied to a marine closed aquaculture system. Water Res.

[5] Zhang H (2023). Novel insights into aerobic denitrifying bacterial communities augmented denitrification capacity and mechanisms in lake waters. Sci Total Environ.

[6] Zhang M (2021). Impact of functional microbes on nitrogen removal in artificial tidal wetlands in the Yangtze River estuary: evidence from molecular and stable isotopic analyses. J Clean Prod.

[7] Zhao X (2023). Microalgae-based constructed wetland system enhances nitrogen removal and reduce carbon emissions: performance and mechanisms. Sci Total Environ.

[8] Han D (2022). Nitrogen removal of water and sediment in grass carp aquaculture ponds by mixed nitrifying and denitrifying bacteria and its effects on bacterial community. Water (Basel).

[9] Wallenstein MD (2006). Environmental controls on denitrifying communities and denitrification rates: insights from molecular methods. Ecol Appl.

[10] Zhi W, Ji G (2014). Quantitative response relationships between nitrogen transformation rates and nitrogen functional genes in a tidal flow constructed wetland under C/N ratio constraints. Water Res.

[11] Castine SA (2012). Denitrification and anammox in tropical aquaculture settlement ponds: an isotope tracer approach for evaluating N2 production. PLoS ONE.

[12] Kuenen JG (2008). Anammox bacteria: from discovery to application. Nat Rev Microbiol.

[13] Broadbent FE, Clark F (1965). Denitrification. Agronomy (Basel).

[14] Reay DS (2012). Global agriculture and nitrous oxide emissions. Nat Clim Chang.

[15] Arias CR (2010). Combined use of 16S ribosomal DNA and automated ribosomal intergenic spacer analysis to study the bacterial community in catfish ponds. Lett Appl Microbiol.

[16] Thomson AJ (2012). Biological sources and sinks of nitrous oxide and strategies to mitigate emissions. Philos Trans.

[17] Bai R (2015). Candidatus Brocadia and Candidatus Kuenenia predominated in anammox bacterial community in selected Chinese paddy soils. J Soils Sediments.

[18] Wang J, Gu JD (2013). Dominance of Candidatus Scalindua species in anammox community revealed in soils with different duration of rice paddy cultivation in Northeast China. Appl Microbiol Biot.

[19] Yang XR (2015). Alterations in anaerobic ammonium oxidation of paddy soil following organic carbon treatment estimated using 13C-DNA stable isotope probing. Appl Microbiol Biotechnol.

[20] Philippot L, Hallin S, Schloter M (2007). Ecology of denitrifying prokaryotes in agricultural soil. Adv Agron.

[21] Schmid MC (2005). Biomarkers for in situ detection of anaerobic ammonium-oxidizing (anammox) bacteria. Appl Environ Microbiol.

[22] Wang Z (2017). Enhancing nitrogen removal via the complete autotrophic nitrogen removal over nitrite process in a modified single-stage tidal flow constructed wetland. Ecol Eng.

[23] Wang Z (2017). Enhanced nitrogen removal and associated microbial characteristics in a modified single-stage tidal flow constructed wetland with step-feeding. Chem Eng J.

[24] Zhang Q (2024). Multiple isotopes reveal the driving mechanism of high NO_3_^-^ level and key processes of nitrogen cycling in the lower reaches of Yellow River. J Environ Sci (China).

[25] Wang M (2022). Nitrogen removal performance, and microbial community structure of water and its association with nitrogen metabolism of an ecological engineering pond aquaculture system. Aquaculture Rep.

[26] Liu Q (2023). Microbial communities and nitrogen cycling in Litopenaeus vannamei and Mercenaria polyculture ponds. Aquaculture Rep.

[27] Hou J (2017). Achieving short-cut nitrification and denitrification in modified intermittently aerated constructed wetland. Biores Technol.

[28] Wang S (2021). Stable nitrogen removal by anammox process after rapid temperature drops: Insights from metagenomics and metaproteomics. Biores Technol.

[29] Zhang M (2020). Nitrogen loss by anaerobic ammonium oxidation in a mangrove wetland of the Zhangjiang Estuary, China. Sci Total Environ.

[30] Bai R (2015). Activity, abundance and community structure of anammox bacteria along depth profiles in three different paddy soils. Soil Biol Biochem.

[31] Li H (2016). The phenological stage of rice growth determines anaerobic ammonium oxidation activity in rhizosphere soil. Soil Biol Biochem.

[32] Tan E (2019). Organic matter decomposition sustains sedimentary nitrogen loss in the Pearl River Estuary, China. Sci Total Environ.

[33] Engstrom P (2005). Anaerobic ammonium oxidation by nitrite (anammox): implications for N2 production in coastal marine sediments. Geochim Cosmochim Acta.

[34] Thamdrup B, Dalsgaard T (2002). Production of N2 through anaerobic ammonium oxidation coupled to nitrate reduction in marine sediments. Appl Environ Microbiol.

[35] Sonthiphand P, Hall MW, Neufeld JD (2014). Biogeography of anaerobic ammonia-oxidizing (anammox) bacteria. Front Microbiol.

[36] Dang H (2013). Molecular detection of Candidatus Scalindua pacifica and environmental responses of sediment anammox bacterial community in the Bohai Sea, China. PLoS ONE.

[37] Humbert S (2010). Molecular detection of anammox bacteria in terrestrial ecosystems: distribution and diversity. ISME J.

[38] Lam P (2009). Revising the nitrogen cycle in the Peruvian oxygen minimum zone. Biol Sci.

[39] Jetten MS (2009). Biochemistry and molecular biology of anammox bacteria. Crit Rev Biochem Mol Biol.

[40] Zhu G (2013). Hotspots of anaerobic ammonium oxidation at lande-freshwater interfaces. Nat Geosci.

[41] Zhao S (2018). Linking abundance and community of microbial N2O-producers and N2O-reducers with enzymatic N2O production potential in a riparian zone. Sci Total Environ.

[42] Han B (2021). Rates and microbial communities of denitrification and anammox across coastal tidal flat lands and inland paddy soils in East China. Appl Soil Ecol.

[43] Ducey TF (2015). Soil physicochemical conditions, denitrification rates, and nosZ abundance in North Carolina Coastal Plain restored wetlands. J Environ Qual.

[44] Philippot L (2009). Mapping field-scale spatial patterns of size and activity of the denitrifier community. Environ Microbiol.

[45] Liu W (2018). Sediment denitrification in Yangtze lakes is mainly influenced by environmental conditions but not biological communities. Sci Total Environ.

[46] Cao S (2019). Novel two stage partial denitrification (PD)-Anammox process for tertiary nitrogen removal from low carbon/nitrogen (C/N) municipal sewage. Chem Eng J.

[47] Chen D (2019). Denitrification- and anammox-dominant simultaneous nitrification, anammox and denitrification (SNAD) process in subsurface flow constructed wetlands. Bioresour Technol.

[48] Liu W (2015). Sediment denitrification and nitrous oxide production in Chinese plateau lakes with varying watershed land uses. Biogeochemistry.

[49] Dalsgaard T, Thamdrup B, Canfield DE (2005). Anaerobic ammonium oxidation (anammox) in the marine environment. Res Microbiol.

[50] Schmidt CS, Richardson DJ, Baggs EM (2011). Constraining the conditions conducive to dissimilatory nitrate reduction to ammonium in temperate arable soils. Soil Biol Biochem.

[51] Tiedje JM (1982). Denitrification: ecological niches, competition and survival. Antonie Van Leeuwenhoek.

[52] Samad MDS (2016). Phylogenetic and functional potential links pH and N_2_O emissions in pasture soils. Sci Rep.

[53] Lage S, Toffolo A, Gentili FG (2021). Microalgal growth, nitrogen uptake and storage, and dissolved oxygen production in a polyculture based-open pond fed with municipal wastewater in northern Sweden. Chemosphere.

